# Impact of green tea on the deleterious cardiometabolic effects of 7‐days unhealthy lifestyle in young healthy males

**DOI:** 10.14814/phy2.14720

**Published:** 2021-03-07

**Authors:** Kirsty A. Roberts, Richard Draijer, Nicola D. Hopkins, Young de Graaf, Sophie M. Holder, Sophie E. Carter, Dick H. J. Thijssen, David A. Low

**Affiliations:** ^1^ Research Institute for Sport and Exercise Science Liverpool John Moores University Liverpool UK; ^2^ Unilever Foods Innovation Centre Wageningen The Netherlands; ^3^ Department of Physiology Research Institute for Health Science Radboud University Nijmegen Medical Center Nijmegen The Netherlands

**Keywords:** cardiometabolic health, cardiovascular disease, flavonoids, overfeeding, physical inactivity

## Abstract

**Purpose:**

The aim of this study was to examine if catechin‐rich green tea abrogates the negative effects of 7‐days of physical inactivity and excessive calorie‐intake on insulin homeostasis and peripheral vascular function.

**Methods:**

Using a randomized, double‐blind, crossover design, twelve healthy men (29 ± 6 yrs) underwent 7‐days unhealthy lifestyle (UL), including physical inactivity (−50% steps/day) and overfeeding (+50% kcal/day). This was combined with green tea consumption (UL‐tea; 3 doses/day) or placebo (UL‐placebo). Before and after each intervention, we examined postprandial blood glucose and insulin (3‐h after a 1,202 kcal meal) and upper and lower limb vascular function (flow‐mediated dilation (FMD%)) and carotid artery reactivity (CAR%).

**Results:**

UL‐placebo increased postprandial glucose and insulin, while UL‐tea decreased postprandial glucose and insulin (Time*Intervention interaction effects: both *p* < 0.05). UL‐placebo decreased CAR% and femoral FMD%, while UL‐tea prevented these effects (Time*Intervention interaction effects of *p* < 0.04 and *p* < 0.001, respectively). There was no main effect of Time or Time*Intervention interaction (both *p* > 0.05) for brachial FMD%.

**Conclusion:**

Seven days of physical inactivity and overfeeding impair insulin homeostasis and vascular function. These effects were mitigated by a daily intake of catechin‐rich green tea.

## Graphical abstract

The aim of this study was to examine if catechin‐rich green tea abrogates the negative effects of short‐term physical inactivity and excessive calorie‐intake on insulin homeostasis and peripheral vascular function. Twelve healthy men (29 ± 6 yrs) underwent 7‐days unhealthy lifestyle (UL), including physical inactivity (−50% steps/day) and overfeeding (+50% kcal/day) combined with green tea consumption (UL‐tea; 3 doses/day) or placebo (UL‐placebo). UL‐placebo increased post‐prandial glucose and insulin, while UL‐tea decreased post‐prandial glucose and insulin. UL‐placebo decreased carotid artery reactivity and lower limb vascular function, while UL‐tea prevented these effects. Seven days of physical inactivity‐ and overfeeding‐induced impairments in insulin homeostasis and vascular function were mitigated by daily intake of catechin‐rich green tea.

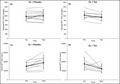

## 1 INTRODUCTION

Physical inactivity and poor dietary habits are major modifiable risk factors linked to detrimental changes in cardiometabolic health (WHO, 2017). Large cohort studies revealed that a physically inactive lifestyle, either classified as the lack of exercise or engagement in sedentary behavior, is strongly associated with increased cardiovascular disease (CVD) risk (Wilmot et al., 2012). Similarly, habitual high (trans) fat and high‐calorie dietary intake is associated with increased cardiovascular risk and development of CVD (Souza et al., (2015)). While the long‐term effects of these behaviors are well‐established, relatively less work has examined whether short periods of an unhealthy (high calories, low physical activity) lifestyle affect cardiometabolic risk. Intermittent periods of unhealthy nutritional and physical activity behavior are frequently experienced, such as during holidays, religious festivals or forced physical inactivity (e.g., hospitalization, injury). Previous work has found that 3–14 days of exposure to physical inactivity and/or overfeeding impairs metabolic and vascular health (Boyle et al., 2013; Hamburg et al., 2007; Knudsen et al., 2012). Exposure to such periods of unhealthy behavior may ultimately contribute to the accelerated development of cardiometabolic disorders; therefore, effective strategies are needed to offset these deleterious effects of a short‐term unhealthy lifestyle.

Dietary interventions are inexpensive tools to combat the ever‐increasing burden of CVD. Bioactive compounds known as polyphenols are found in plant‐derived products, such as olive oil, fruits, and vegetables and are suggested to be cardioprotective and exert a positive influence upon cardiovascular health (Mangels & Mohler, 2017). Polyphenols are the most abundant antioxidant in the human diet and can be broadly categorized into four subclasses: flavonoids, phenolic acids, lignans, and stilbenes. Flavonoids account for the greatest proportion of polyphenols (60%) and have been linked to a reduction in CVD risk (Kim et al., 2016; Ponzo et al., 2015). Tea is the major source of dietary flavonoids in many countries globally (Yahya et al., 2016) and is classified according to the fermentation process, where flavonoids present in the tea leaf are oxidized following the release of intracellular polyphenol oxidase. The four major types of tea are white tea, green tea (non‐fermented), oolong tea (semi‐fermented), and black tea (fully fermented). The associated health benefits of green tea are attributed to its richness in flavan‐3‐ols (catechins) (Hodgson & Croft, 2010). The main catechins present in green tea are epicatechin (EC), epigallocatechin (EGC), epicatechin‐3‐gallate (ECG), and epigallocatechin‐3‐gallate (EGCG), the most abundant of which is EGCG (~59%) followed by EGC (~19%), ECG (~14%) and EC (~6%) (Cabrera et al., 2006).

Several biological actions of green tea support the association with a cardioprotective effect, with a direct impact of tea on the vasculature, including its effects on the vascular endothelium (Grassi, Desideri, et al., 2009), the inner lining of all blood vessels which plays a central role in vascular homeostasis, and improving the bioactivity of NO (Grassi et al., 2013). Furthermore, higher green tea consumption is associated with lower blood pressure (Peng et al., 2014) and superior endothelial function (Alexopoulos et al., 2008; Jochmann et al., 2008), particularly in those with CVD or in the postprandial state (Corretti et al., 2002; Park et al., 2010; Ras et al., 2011). Clinically, green tea ingestion is also linked to lower risk for CVD events and cerebrovascular complications (e.g., stroke, dementia) (Commenges et al., 2000; Vita, 2005). In addition, regular intake of tea, a key dietary source of flavonoids, is associated with lower risk for type 2 diabetes mellitus (Jing et al., 2009; Park et al., 2014). In support of this, some laboratory‐based studies have found tea to acutely improve glucose homeostasis in both healthy (Wu et al., 2012), diabetic and obese individuals (Bogdanski et al., 2012; Liu et al., 2014; Nagao et al., 2009). The consumption of catechin‐rich green tea against a background of forced physical inactivity and overfeeding could mitigate the negative metabolic and vascular effects of physical inactivity and overfeeding, at least in the short‐term. Therefore, in this study, we tested the hypothesis that the daily consumption of green tea abrogates the effects of 7‐days unhealthy lifestyle (UL: 50% less physical activity and 50% more calories) on glucose‐insulin homeostasis and vascular function in healthy participants.

## 2 PARTICIPANTS AND METHODS

### 2.1 Participants

Fourteen healthy, non‐smoking, habitually active male participants were recruited through local advertisement (29 ± 6 yrs, BMI 25 ± 2 kg/m^2^ and mean arterial pressure 84 ± 8 mmHg). This sample size (effect size of 0.9, beta = 0.90, alpha = 0.05) was based on previously reported green tea‐induced increases in macrovascular function (Alexopoulos et al., 2008; Jochmann et al., 2008; Park et al., 2010) and amelioration of fat loading‐induced decrements in macrovascular function (Corretti et al., 2002). We excluded individuals with vasoactive medications, a history of hypercholesterolemia (cholesterol >6.5 mmol/l), CVD, and/or hypertension (systolic: ≥140 mmHg, diastolic: ≥90 mmHg). We also excluded individuals with food allergies, special dietary requirements, currently following a diet, and/or those using dietary/vitamin supplements. Nine participants were habitual users of tea (and coffee). We included physically active individuals [i.e., >8,000 steps/day; (Tudor‐Locke et al., 2011)]. Prior to testing, fully informed written consent was obtained. The study conformed to the *Declaration of Helsinki*, was approved by Liverpool John Moores University's Research Ethics Committee (15/SPS/065) and was registered online (clinicaltrials.gov: NCT02777853).

### 2.2 Experimental design

First, participants underwent a 4‐day monitoring period to record physical activity level and dietary intake. Subsequently, participants underwent a randomized double‐blind, placebo‐controlled, crossover trial design of 5 weeks duration; lead‐in period (1 week); intervention period one (1 week); washout (2 weeks); and finally intervention period two (1 week). A 2 week washout period was used to allow the systemic elimination of the tea and unhealthy lifestyle before the initiation of the subsequent 1‐week intervention which was based on previous short‐term studies that demonstrated detrimental effects of forced physical inactivity and/or overfeeding interventions on insulin sensitivity and macrovascular function (Boyle et al., 2013; Hagobian & Braun, 2006; Knudsen et al., 2012; Parry et al., 2017; Walhin et al., 2013). Participants adopted an unhealthy lifestyle in both intervention periods but were randomly assigned (computer‐generated, simple randomization), to tea (UL‐Tea) in intervention period one, followed by placebo (UL‐Placebo) in intervention period two, or placebo in intervention period one followed by tea in intervention period two. A crossover design was chosen for this study instead of the more traditional randomized, parallel‐group design because within‐participant variation is less than between‐participant variation allowing for the examination of possible causal relationships between the interventions (green tea vs. placebo) and the outcomes.

### 2.3 Interventions

#### 2.3.1 Unhealthy Lifestyle (UL)

Based on the 4‐day control period, participants reduced daily steps by 50%. Real‐time feedback on step count was provided using a pedometer (Digi‐walker SW‐701, Yamax) and verified post hoc via a hip‐mounted accelerometer (GT3X BT+model, Actigraphy). During the interventions daily caloric intake was increased by 50% (overfeeding) through the provision of daily “snack boxes” in addition to participants maintaining their normal diet. The snack boxes were made up of 60% and 20% of fats and carbohydrates, respectively, and typically contained foods such as cheddar cheese, whole milk, salami, eggs, white chocolate, and croissants. The participants’ baseline dietary ratios of macronutrients were 49% carbohydrates, 31% fat, and 20% protein. Participants also refrained from foods and beverages high in flavonoids (e.g., berries, red wine, dark chocolate) and caffeine during both interventions. Dietary patterns were monitored and analyzed (MyFitnessPal, Baltimore, Maryland, USA) through self‐reported food diaries. Step count verification was performed using accelerometry data (ActiLife 6).

#### 2.3.2 Tea versus placebo

Participants drank three doses of green tea (UL‐Tea, Unilever) or placebo (UL‐Placebo) per day >15‐min before breakfast, lunch, and dinner. In a double‐blind manner, tea was provided as a brewed spray‐dried tea powder form, supplied in identical, coded, laminated aluminium foil sachets. Two sachets were dissolved in 300 ml of boiled water. No additives were permitted and tea was consumed while hot. This dose of green tea is estimated to contain ~300 mg of flavonoids (Astill et al., 2001). Due to a difference in energy intake between green tea and placebo because of maltodextrin in the green tea (19 kcal/day), daily energy intake was adjusted for in the daily food intake. Placebo tea had similar color and taste like green tea, but did not contain flavonoids or caffeine (Table S1; https://figshare.com/s/8831f983188aba13d264). Participants were instructed to avoid all other types of tea.

### 2.4 Experimental measures

Participants reported to the laboratory before and after each 7‐day intervention. In the week preceding the pre‐intervention visits, participants refrained from tea and avoided food sources high in flavonoids (Perez‐Jimenez et al., 2010). Prior to testing, participants fasted for >6‐h and refrained from alcohol and strenuous physical activity for 24 h. Measurements were conducted in a quiet, temperature‐controlled laboratory (22–24°C) at the same time of day. Upon arrival, anthropometric measurements were recorded, including height (Seca stadiometer, model 217, Birmingham, UK) and body mass (Seca, model 767, Germany). Before and after each intervention, we examined vascular function and glucose homeostasis/insulin sensitivity responses to a mixed meal tolerance test Assessments of vascular function were always conducted first followed by the mixed meal tolerance test.

### 2.5 Mixed meal tolerance test

A 20G cannula (Venflon Pro) was inserted into the antecubital vein of one arm and a three‐way stopcock (BD Connecta) was subsequently attached to enable multiple venous blood sampling and flushing of the cannula. Baseline samples were collected for glucose (5 ml) and insulin (6 ml), in silica and EDTA vacutainers, respectively. After baseline assessment, participants consumed a mixed meal (1201 kcal, comprising 60% carbohydrates, 33% fat, and 7% protein; Table S2; https://figshare.com/s/8831f983188aba13d264; https://doi.org/10.6084/m9.figshare.12246035) in ~15 min (Marena et al., 1992). Postprandial blood samples were collected after 30, 60, 90, 120, and 180 min. The rationale for using a 180 min postprandial period was in order to ensure peak responses and subsequent declines in glucose and insulin were detected as well as previous work that has demonstrated black tea‐induced beneficial vascular and insulin effects for 180 min after a mixed‐meal challenge (Fuchs et al., 2016). Following each blood sample, isotonic saline (3 ml; B Braun, UK) was used to keep the cannula patent. All blood samples were centrifuged (1,000 g for 10 min at 4°C) to obtain plasma samples, which were subsequently stored in aliquots at −80°C for later analysis using commercially available assays for glucose (Randox, London, UK) and insulin (ELISA‐kit, Invitrogen). Plasma glucose was determined using an ILab‐600 semi‐automatic spectrophotometric analyzer and glucose hexokinase assay (Randox). Plasma insulin concentrations were determined using a direct insulin ELISA kit (Invitrogen) and insulin levels were determined using a monochromator microplate reader (CLARIOstar, BMG LABTECH, Ortenberg). Area‐under‐the‐curve (AUCs) for postprandial glucose and insulin were calculated above baseline using the trapezoidal rule.

Insulin sensitivity was estimated using homeostasis model assessment (HOMA‐IR) (Hanson et al., 2000) and insulin secretion from insulin and glucose levels obtained following the standard meal challenge using the Matsuda index (Matsuda & DeFronzo, 1999). β‐Cell function was assessed with the oral disposition index (DIo) (Utzschneider et al., 2009).

### 2.6 Vascular function

Peripheral conduit artery, largely NO‐mediated, endothelial function was examined at the right brachial and superficial femoral arteries using flow‐mediated dilation (FMD) (Thijssen et al., 2011). A 10 MHz multi‐frequency linear array probe, attached to a high‐resolution 2D duplex ultrasound machine (Terason uSmart 3300, Teratech) was used. Pneumatic cuffs (D.E. Hokanson), connected to a rapid inflator (D.E. Hokanson), were positioned on the interrogated upper forearm and thigh, distal to the imaged site. In addition to a stable B‐mode image, continuous Doppler velocity and diameter data were collected. Baseline images were recorded for 1 min, following which the occlusion cuffs were inflated (>220 mmHg) for 5 min. Diameter and velocity recordings resumed 30 seconds prior to cuff deflation and continued for 3 min after cuff deflation, according to methodological guidelines (Thijssen et al., 2011).

Central conduit artery endothelial function was measured using the carotid artery reactivity test (CAR). The CAR induces carotid artery dilation during sympathetic stimulation using the cold pressor test (CPT) and is a surrogate for coronary artery vasomotor function and is inversely associated with the presence of cardiovascular risk factors (Mil et al., 2017; Rubenfire et al., 2000). Duplex ultrasound was used to examine the common carotid artery (CCA) before (1 min) and during the CPT when participants were instructed to immerse their left hand (up to the wrist) in iced slush (1–5°C) for 3 min. Participants were instructed to breathe normally throughout the CPT and to avoid breath‐holding/hyperventilation. Beat‐to‐beat arterial BP (Finapres Medical Systems) and 5‐lead ECG were recorded online throughout the CPT (LabChart 8.0, AD Instruments). Baseline diameter, velocity, shear rate, and blood flow were calculated as the mean of data acquired across the 1 min preceding the CPT and during the CPT, data were calculated as the mean value for 10‐second intervals for the 3 min (Mil et al., 2017).

FMD and CAR analysis was performed using custom‐designed edge detection software by a single trained researcher who was blinded to the treatment allocation (Thijssen et al., 2011). From the synchronized diameter and velocity data, blood flow (the product of cross‐sectional area and Doppler velocity) and shear rate (four times the velocity divided by the diameter) were calculated. Total shear rate area under the curve between cuff deflation and peak diameter (SRAUC) was calculated and FMD and CAR were automatically calculated and presented as the peak diameter change from baseline (in %). The area under the curve for changes in diameter during the CPT (CARAUC) was calculated as the percent change in the average carotid diameter during the 3‐min CPT from baseline. As part of the complete study (clinicaltrials.gov: NCT02777853), we also examined microvascular function via the assessment of forearm skin blood flow responses to local skin heating. However, due to space restrictions and this variable being a secondary outcome, these data are only presented as supplements (https://figshare.com/s/ee9578ba1100e868861f; https://doi.org/10.6084/m9.figshare.12659987).

### 2.7 Statistical analysis

Data were expressed as mean ± SD and statistical significance was set at *p* < 0.05. Linear mixed models were used to examine the effect of the 7‐day intervention (“Time”: pre vs. post), and whether this effect was altered by the type of intervention (“Intervention”: Placebo vs. Tea). The repeated covariance type was Unstructured, while we specified”Time,” “Intervention,” and “Time*Intervention” as Fixed Effects (intercept was included) and as Estimated Marginal Means. Significant main or interaction effects were followed up with the least significant difference (LSD) approach to multiple comparisons (Perneger, 1998). Data were analyzed using SPSS 22.0 (SPSS).

## 3 RESULTS

Two participants withdrew prior to completion due to personal circumstances (n = 1) and being unable to tolerate the lifestyle change (n = 1), while technical issues caused incomplete data sets for some parameters. One participant was unable to complete the cold pressor test due to discomfort (n = 11). Due to problems with venous cannulation, one participant did not complete measures of glucose handling and insulin homeostasis (n = 11). Self‐reported compliance with tea and food boxes was 100%. Compared to baseline (11,103 ± 3,385 steps/day), a significant reduction in steps was found after UL‐Placebo (5,880 ± 1,462 steps/day, *p* < 0.001) and UL‐Tea (5,710 ± 1,390 steps/day, *p* < 0.001) with no difference between UL‐Placebo and UL‐Tea (*p* = 0.75). Energy intake increased during both UL‐Placebo (3,519 ± 1,279 kcal/day) and UL‐Tea (3,516 ± 1,210 kcal/day) compared to baseline (2,373 ± 864 kcal/day, both *p* < 0.001) with no difference between UL‐Placebo and UL‐Tea (*p* = 0.95). A non‐significant increase in body mass was found in UL‐Placebo (77.4 ± 10.0 to 78.1 ± 11.0 kg) and UL‐Tea (76.9 ± 9.0 to 77.6 ± 10.6 kg, *p* = 0.07), which did not differ between interventions (“Time*Intervention”‐interaction: *p* = 0.92). A trend for a “Time*Intervention” interaction was found for MAP (*p* = 0.06), with small, non‐significant changes in the opposite direction after UL‐Placebo (83 ± 5 vs. 85 ± 5 mmHg) and UL‐tea (84 ± 7 vs. 82 ± 6 mmHg).

### 3.1 Mixed‐Meal Tolerance Test (MTT)

**FIGURE 1:**
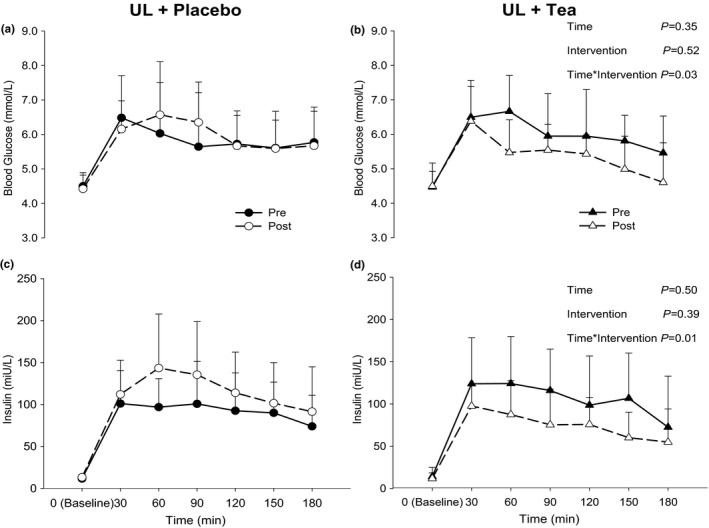
Presentation of glucose (a‐b) and insulin (c‐D) levels at baseline (0 min) and after a mixed meal tolerance test (MTT; 30, 60, 90, 120, 150, and 180 min) before (closed symbols) and after (open symbols) a 7‐day unhealthy lifestyle (UL) combined with placebo (a, c) or green tea (b, d) in healthy male volunteers. Data are presented as means, with error bars representing SD. *P*‐values refer to a 2‐way linear mixed model (LMM) of time and intervention. N = 11

**FIGURE 2:**
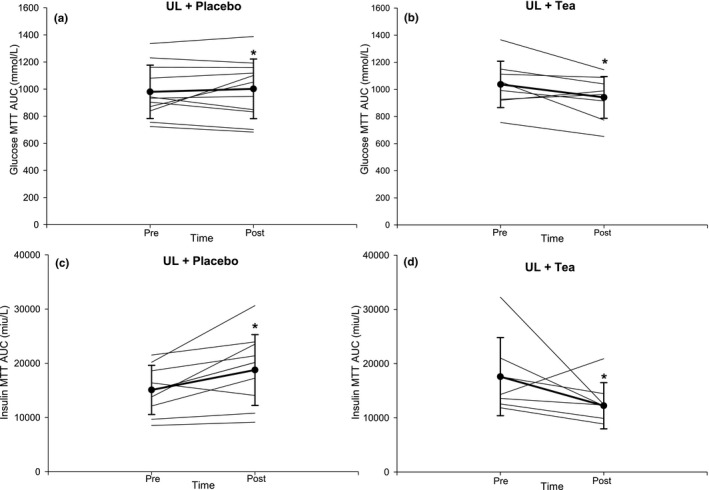
Presentation of individual and mean glucose (a‐b) and insulin (c‐d) AUC responses to a mixed meal tolerance test (MTT) before and after a 7‐day unhealthy lifestyle combined with placebo (a, c) or green tea (b, d) in healthy male volunteers. Error bars represent SD. N = 11. *p < 0.05 versus Pre

The 3‐h mixed‐meal tolerance (MTT) induced a typical initial increase and subsequent decrease in glucose and insulin (Figure 1). A significant “Time*Intervention” interaction effect was found for glucose and insulin (*p* = 0.03 and 0.01, respectively, Figure 1). Post hoc analysis revealed that postprandial AUC for glucose (226 ± 138 vs. 261 ± 162 mmol/L) and insulin (12,562 ± 4,498 vs. 16,254 ± 6,803 miu/L) were increased in UL‐Placebo (both *p* < 0.05), while postprandial AUC for glucose (261 ± 120 vs. 164 ± 113 mmol/L) and insulin (15,225 ± 5,501 vs. 10,533 ± 3,825 miu/L) were significantly decreased in UL‐Tea (both *p* < 0.05; Figure 2). There was a significant “Time*Intervention” interaction (*p* = 0.01) for the Matsuda Index responses with a reduction after UL‐Placebo (3.7 ± 2.0 vs. 3.0 ± 1.3, *p* < 0.05) but no change after UL‐Tea (3.3 ± 1.7 vs. 4.2 ± 2.2, *p* > 0.05). There was no significant “Time*Intervention” interaction (*p* = 0.53) for the HOMA‐IR responses with no change after either UL‐Placebo (2.4 ± 1.2 vs. 2.6 ± 0.5) or UL‐Tea (2.8 ± 2.1 vs. 2.5 ± 1.8). There was no significant “Time*Intervention” interaction (*p* = 0.11) for the β‐Cell function responses with no change after either UL‐Placebo (9.2 ± 10.2 vs. 6.0 ± 4.7) or UL‐Tea (6.4 ± 5.2 vs. 8.5 ± 6.4).

### 3.2 Peripheral vascular function

**FIGURE 3:**
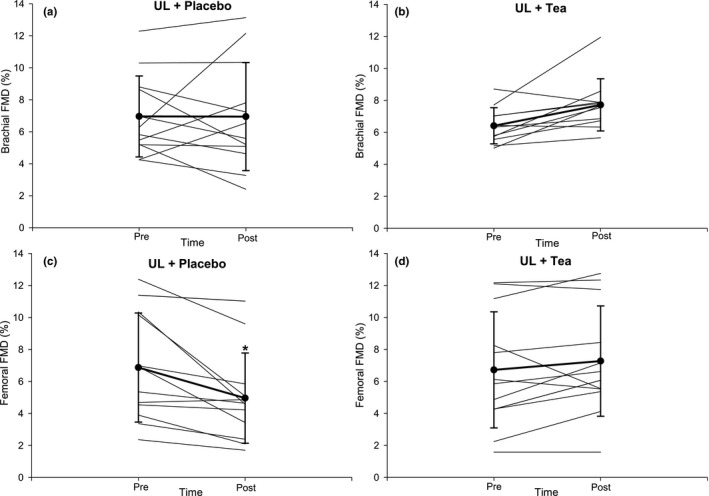
Presentation of individual and mean brachial (a‐b) and femoral (c‐d) FMD responses before and after a 7‐day unhealthy lifestyle combined with placebo (a, c) or green tea (b, d) in healthy male volunteers. Error bars represent SD. N = 11. *p < 0.05 versus Pre

For the brachial artery, there was no main effect of “Time,” “Intervention” or “Time*Intervention” interaction for FMD%, baseline diameter or SRAUC (all *p* > 0.05, Table 1; Figure 3). For femoral artery FMD, there was a significant interaction of “Time*Intervention” (*p* < 0.001). Post hoc analysis revealed that femoral artery FMD decreased after UL‐Placebo (e.g., peripheral vascular function was worse), but was maintained during UL‐Tea (e.g., peripheral vascular function did not change; Table 1, Figure 3). No effects were observed for baseline diameter or SRAUC (all *p* > 0.05, Table 1). No significant main effects of time nor time*interaction effects were found for skin microvascular function (https://figshare.com/s/ee9578ba1100e868861f; https://doi.org/10.6084/m9.figshare.12659987; Table S3 and Figures S1 and S2).

**TABLE 1:** Brachial and femoral artery FMD%, baseline diameter, time‐to‐peak and shear rate, and carotid artery reactivity variables before and after UL‐Placebo and UL‐Tea interventions. N = 12 for Brachial and Femoral Artery data. N = 11 for Carotid Artery Reactivity data

Brachial artery	Intervention (mean ± *SD*)				LMM *p* values		
UL‐Placebo		UL‐Tea		Time	Intervention	T*I
Pre	Post	Pre	Post			
FMD (%)	7.0 ± 2.5	7.0 ± 3.4	7.0 ± 1.2	7.7 ± 1.6	0.20	0.97	0.11
Baseline diameter (cm)	0.4 ± 0.0	0.4 ± 0.0	0.4 ± 0.0	0.4 ± 0.0	0.40	0.45	0.21
Time‐to‐peak (s)	40 ± 17	48 ± 22	47 ± 19	43 ± 11	0.65	0.87	0.06
Shear rate (SRAUC)	17456 ± 8205	19407 ± 9026	21046 ± 7317	21411 ± 12650	0.64	0.46	0.16
Femoral Artery							
FMD (%)	7.0 ± 3.4	5.0 ± 2.8	6.7 ± 3.6	7.3 ± 3.5	0.10	0.21	0.001
Baseline diameter (cm)	0.6 ± 0.1	0.6 ± 0.1	0.6 ± 0.1	0.7 ± 0.1	0.52	0.29	0.32
Time‐to‐peak (s)	74 ± 49	79 ± 43	54 ± 28	41 ± 22	0.77	0.02	0.30
Shear rate (SRAUC)	17882 ± 8353	18187 ± 13450	15904 ± 8525	13659 ± 7965	0.68	0.15	0.16
Carotid artery reactivity							
CAR (%)	5.1 ± 1.5	3.3 ± 4.3	5.7 ± 5.3	7.5 ± 4.0	0.87	0.05	0.04
CARAUC (%)	2.6 ± 1.5	1.7 ± 2.2	3.4 ± 3.4	4.2 ± 3.0	0.88	0.04	0.08
Change in SBP (mmHg)	28 ± 13	34 ± 15	25 ± 12	26 ± 15	0.27	0.10	0.38
Change in DBP (mmHg)	18 ± 6	22 ± 8	18 ± 5	14 ± 5	0.61	0.07	0.05

Data are mean ± SD.

Abbreviations: AUC, area‐under‐the‐curve; CAR, carotid artery reactivity; DBP; diastolic blood pressure; FMD, flow‐mediated dilation; SBP, systolic blood pressure; SRAUC, shear rate area‐under‐the‐curve; T*I, Time*Intervention‐interaction.

### 3.3 Central vascular function

**FIGURE 4:**
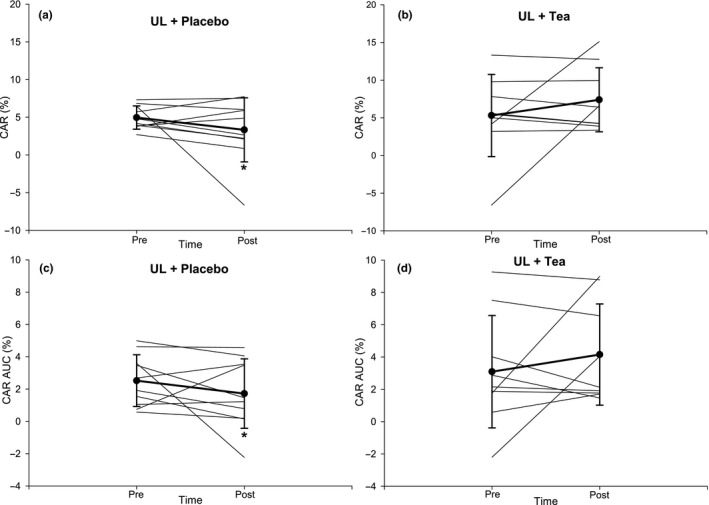
Presentation of individual and mean CAR (a‐b) and CARAUC (c‐d) responses before and after a 7‐day unhealthy lifestyle combined with placebo (a, c) or green tea (b, d) in healthy male volunteers. Error bars represent SD. N = 11. *p < 0.05 versus Pre

For CAR (peak diameter change from baseline), there was no main effect of “Time” (*p* = 0.85), but there was a main effect of “Intervention” (*p* = 0.05) and “Time*Intervention” (*p* = 0.04). Post hoc analysis showed that CAR decreased following UL‐Placebo (e.g., central vascular function was worse), but was maintained during UL‐Tea (e.g., central vascular function did not change; Table 1, Figure 4). Similar results were evident for CARAUC (the percent change in the average carotid diameter during the 3‐min CPT); there was no main effect of “Time” (*p* = 0.88), but there was a main effect of “Intervention” (*p* = 0.04) and a borderline “Time*Intervention” interaction (*p* = 0.08). Post hoc analysis showed that CARAUC decreased following UL‐Placebo, but was maintained during UL‐Tea (Figure 4). Elevations in systolic and diastolic BP during CAR were not different across “Time,” “Intervention” or “Time*Intervention” (all *p* > 0.05, Table 1). Baseline common carotid artery diameter did not change after either intervention (*p* = 0.59) or differed between conditions (*p* = 0.97).

## 4 DISCUSSION

Our study has the following novel observations. Impairments in postprandial glucose‐insulin homeostasis, and also peripheral and central vascular function, in young, healthy men as a result of a 7‐day unhealthy lifestyle, were ameliorated with daily consumption of green tea. These results highlight the rapid, detrimental impact of short‐term exposure to an unhealthy lifestyle on metabolic and vascular function, and that green tea consumption may (in part) alleviate these effects. This work highlights the immediate impact of lifestyle‐related factors on metabolic and cardiovascular health.

In the present study, we found higher blood glucose and insulin levels after a mixed‐meal challenge (as well as a lower Matsuda Index) after 7 days following an unhealthy lifestyle. This supports previous findings, in that 3–14 days exposure to excessive calorie intake, physical inactivity or both can alter glucose and insulin homeostasis (Boyle et al., 2013; Hagobian & Braun, 2006; Knudsen et al., 2012; Parry et al., 2017; Walhin et al., 2013). These findings are clinically relevant since higher postprandial levels of blood glucose and insulin fit with the presence of insulin resistance. More importantly, when meals were consistently preceded with green tea, we found that these metabolic derangements did not occur. Previous studies found that green tea acutely, that is, within hours, improves glucose homeostasis in healthy and pre‐diabetic participants (Bogdanski et al., 2012; Liu et al., 2014; Nagao et al., 2009; Wu et al., 2012). In addition, long‐term ingestion of green tea has been linked to better metabolic health through a range of mechanisms, including, a slowing of carbohydrate digestion and glucose absorption, stimulation of insulin secretion, a decreased β‐cell oxidative damage, a modulation of liver glucose release and activation of glucose uptake receptors in insulin‐sensitive tissue (Guasch‐Ferré et al., 2017). Our study extends these findings by revealing that green tea is causally linked to the prevention of impairments in metabolic function in response to short‐term exposure to an unhealthy lifestyle. We found significant impairments in peripheral and central vascular function, specifically, conduit artery vasodilator capacity to increases in flow, that is, femoral FMD, largely NO‐mediated (Kooijman et al., 2008), was reduced by ~2% points and sympathetic stimulation, that is, CAR, likely related to NO (Peace et al., 2018), was reduced by ~1.8% points after 7 days of unhealthy lifestyle, which did not occur with concomitant consumption of green tea. Meta‐analyses indicate an 8–13% lower risk of CV events per percent point increase in FMD (Thijssen et al., 2019) and a 2% lower CAR is associated with the presence of two CVD risk factors (Mil et al., 2017). Several previous studies found that a prolonged and/or extreme unhealthy lifestyle, for example, diets high in fat (particularly trans‐fat) and/or carbohydrate and/or physical inactivity, is associated with increased CVD risk (Michas et al., 2014; Skeaff & Miller, 2009; Souza et al., 2015), and impaired macrovascular function (Dow et al., 2015; Nosova et al., 2014) largely attributed to endothelial dysfunction from increased oxidative stress and reduced NO bioavailability (Bae et al., 2001). Our study, reflecting a real‐world situation, that is, holidays, further highlights that only a short timeframe, for example, 7 days, is sufficient to induce clinically meaningful detrimental vascular effects, which were abrogated by regular daily consumption of green tea, likely via improved activation of eNOS (Loke et al., 2010) and NO‐mediated endothelial function (Ras et al., 2011), reduced oxidative stress (Bogdanski et al., 2012) and/or an improved antioxidant and anti‐inflammatory capacity (Suliburska et al., 2012). The exact constituent of green tea that causes these beneficial vascular and metabolic effects in vivo is not clear. Equivocal evidence exists for the role of EGCG (Lorenz et al., 2017; Widlansky et al., 2007) and EC (Dower et al., 2015; Schroeter et al., 2006) and caffeine (Buscemi et al., 2010; Tinahones et al., 2008) in green tea‐induced elevations in macrovascular function. Further research is needed to identify the mechanism(s) that underlie cardiovascular and metabolic benefits of green tea.

We found distinct effects in upper and lower limb FMD responses whereby divergent changes were evident in femoral FMD (decreases in Placebo but maintenance in Tea) but not in brachial FMD. This between‐limb discrepancy may relate to differences in activity level across the intervention period, in that our intervention reduced activity of the lower limbs, but not necessarily upper limbs. This may underlie the decline in femoral artery FMD, with preserved brachial FMD. In agreement, previous studies adopting models of physical inactivity affecting lower limbs (e.g., bed rest, lower limb suspension, step reduction) also report a decline in lower limb FMD, with preserved brachial artery FMD (Boyle et al., 2013; Hamburg et al., 2007) as shear stress is reduced in the lower limb but likely preserved in the upper limb where movement is not restricted. Furthermore, the lower limb vessels appear more vulnerable to dysfunction and disease than upper limb vascular beds (Fukudome et al., 1997; Restaino et al., 2015). Similarly, the lack of a time*condition interaction for forearm microvascular function is consistent with the aforementioned regional FMD differences and/or differences in susceptibility for dysfunction in the micro‐ versus macrovasculature.

### 4.1 Limitations

Although relatively modest sample size was included, our study was sufficiently powered to demonstrate a significant impact of an unhealthy lifestyle and tea via a strong methodological design (i.e., double‐blind, within‐subjects cross‐over) across a variety of outcomes from a comprehensive test protocol. It was not possible to ascribe the detriments in vascular and metabolic function specifically to low physical activity or overfeeding per se; this was beyond the scope of the study. Another limitation is that we adopted self‐reported diaries to assess participants’ compliance with the caloric intervention which may be subject to reporting bias. Moreover, we did not determine if the dose and frequency of green tea were sufficient to raise the plasma NO bioavailability and whether alternative mechanisms were evident (e.g., interaction with the gut microbiome). Only young, healthy men were studied, which limits the findings to this cohort. Clearly, female reproductive hormones in premenopausal women, as well as postmenopausal status, can alter vascular function. The interaction of an unhealthy diet and physically inactive lifestyle and the reproductive cycle is an important area that requires further investigation. Similarly, the beneficial effects of flavonoids are more evident in diseased or at‐risk populations; therefore, it is possible that green tea would have a greater effect in groups with impaired vascular and/or metabolic function. Finally, green tea was used as the intervention when various other types of tea are available, for example, black tea, which shows similar beneficial effects to green tea on vascular and metabolic function (Bryans et al., 2007; Grassi, Mulder, et al., 2009).

### 4.2 Conclusion

In conclusion, our study reveals that only 7 days of an unhealthy lifestyle, including 50% fewer steps and 50% more calories, leads to impaired postprandial metabolic, as well as peripheral and central vascular, function in young, healthy men. These short‐term detrimental metabolic and vascular effects were prevented when green tea was consumed daily. This suggests that simple dietary adjustments, such as the consumption of green tea, may help to avoid short‐term detrimental effects when healthy participants are transiently exposed to an unhealthy lifestyle.

## AUTHOR CONTRIBUTIONS

Experiments were conducted in the Cardiovascular laboratories of the Research Institute for Sport and Exercise Sciences at Liverpool John Moores University. KAR, RD, NDH, DHJT, and DAL designed the work; KAR, SMH, SEC, YdG, and DAL were responsible for the acquisition, analysis, and/or interpretation of the data for the work. KAR, RD, NDH, DHJT, and DAL were responsible for drafting the work or revising it critically for important intellectual content. All authors approved the final version of the manuscript.

## References

[phy214720-bib-0001] Alexopoulos, N. , Vlachopoulos, C. , Aznaouridis, K. , Baou, K. , Vasiliadou, C. , Pietri, P. , Xaplanteris, P. , Stefanadi, E. , & Stefanadis, C. (2008). The acute effect of green tea consumption on endothelial function in healthy individuals. European Journal of Cardiovascular Prevention and Rehabilitation, 15, 300–305. 18525384 10.1097/HJR.0b013e3282f4832f

[phy214720-bib-0002] Astill, C. , Birch, M. R. , Dacombe, C. , Humphrey, P. G. , & Martin, P. T. (2001). Factors affecting the caffeine and polyphenol contents of black and green tea infusions. Journal of Agricultural and Food Chemistry, 49, 5340–5347. 11714326 10.1021/jf010759+

[phy214720-bib-0003] Bae, J.‐H. , Bassenge, E. , Kim, K.‐B. , Kim, Y.‐N. , Kim, K.‐S. , Lee, H.‐J. , Moon, K.‐C. , Lee, M.‐S. , Park, K.‐Y. , & Schwemmer, M. (2001). Postprandial hypertriglyceridemia impairs endothelial function by enhanced oxidant stress. Atherosclerosis, 155, 517–523. 11254924 10.1016/s0021-9150(00)00601-8

[phy214720-bib-0004] Bogdanski, P. , Suliburska, J. , Szulinska, M. , Stepien, M. , Pupek‐Musialik, D. , & Jablecka, A. (2012). Green tea extract reduces blood pressure, inflammatory biomarkers, and oxidative stress and improves parameters associated with insulin resistance in obese, hypertensive patients. Nutrition Research, 32, 421–427. 22749178 10.1016/j.nutres.2012.05.007

[phy214720-bib-0005] Boyle, L. J. , Credeur, D. P. , Jenkins, N. T. , Padilla, J. , Leidy, H. J. , Thyfault, J. P. , & Fadel, P. J. (2013). Impact of reduced daily physical activity on conduit artery flow‐mediated dilation and circulating endothelial microparticles. Journal of Applied Physiology, 115(1519–1525), 2013. 10.1152/japplphysiol.00837.2013 PMC3841822 24072406

[phy214720-bib-0006] Bryans, J. A. , Judd, P. A. , & Ellis, P. R. (2007). The effect of consuming instant black tea on postprandial plasma glucose and insulin concentrations in healthy humans. Journal of the American College of Nutrition, 26, 471–477. 17914136 10.1080/07315724.2007.10719638

[phy214720-bib-0007] Buscemi, S. , Verga, S. , Batsis, J. A. , Donatelli, M. , Tranchina, M. R. , Belmonte, S. , Mattina, A. , Re, A. , & Cerasola, G. (2010). Acute effects of coffee on endothelial function in healthy subjects. European Journal of Clinical Nutrition, 64, 483–489. 20125186 10.1038/ejcn.2010.9

[phy214720-bib-0008] Cabrera, C. , Artacho, R. , & Gimenez, R. (2006). Beneficial effects of green tea ‐ A review. Journal of the American College of Nutrition, 25, 79–99. 16582024 10.1080/07315724.2006.10719518

[phy214720-bib-0009] Commenges, D. , Scotet, V. , Renaud, S. , Jacqmin‐Gadda, H. , Barberger‐Gateau, P. , & Dartigues, J. F. (2000). Intake of flavonoids and risk of dementia. European Journal of Epidemiology, 16, 357–363. 10959944 10.1023/a:1007614613771

[phy214720-bib-0010] Corretti, M. C. , DeLeon, Y. W. , Mangano, C. , Vogel, R. A. , & Plotnick, G. D. (2002). The acute effect of tea consumption with a high fat meal on flow‐mediated brachial artery vasodilation in healthy individuals. Journal of the American College of Cardiology. 39(Suppl 1), 218.

[phy214720-bib-0011] de Souza, R. J. , Mente, A. , Maroleanu, A. , Cozma, A. I. , Ha, V. , Kishibe, T. , Uleryk, E. , Budylowski, P. , Schünemann, H. , Beyene, J. , & Anand, S. S. (2015). Intake of saturated and trans unsaturated fatty acids and risk of all cause mortality, cardiovascular disease, and type 2 diabetes: systematic review and meta‐analysis of observational studies. British Medical Journal, 351, h3978. 26268692 10.1136/bmj.h3978 PMC4532752

[phy214720-bib-0012] Dow, C. A. , Stauffer, B. L. , Greiner, J. J. , & DeSouza, C. A. (2015). Influence of habitual high dietary fat intake on endothelium‐dependent vasodilation. Applied Physiology, Nutrition & Metabolism, 40, 711–715. 10.1139/apnm-2015-0006 PMC4864433 26058441

[phy214720-bib-0013] Dower, J. I. , Geleijnse, J. M. , Gijsbers, L. , Zock, P. L. , Kromhout, D. , & Hollman, P. C. (2015). Effects of the pure flavonoids epicatechin and quercetin on vascular function and cardiometabolic health: a randomized, double‐blind, placebo‐controlled, crossover trial. The American Journal of Clinical Nutrition, 101, 914–921. 25934864 10.3945/ajcn.114.098590

[phy214720-bib-0014] Fuchs, D. , Nyakayiru, J. , Draijer, R. , Mulder, T. P. , Hopman, M. T. , Eijsvogels, T. M. , & Thijssen, D. H. (2016). Impact of flavonoid‐rich black tea and beetroot juice on postprandial peripheral vascular resistance and glucose homeostasis in obese, insulin‐resistant men: a randomized controlled trial. Nutrition & Metabolism, 13, 34. 27182277 10.1186/s12986-016-0094-x PMC4866334

[phy214720-bib-0015] Fukudome, Y. , Fujii, K. , Abe, I. , Ohya, Y. , Fukuhara, M. , Kaseda, S. , Onaka, U. , Tsuchihashi, T. , & Fujishima, M. (1997). Ultrasonographic assessment of regional differences in atherosclerotic lesions in patients with hypertension, diabetes mellitus, or both. Hypertension Research, 20, 175–181. 9328798 10.1291/hypres.20.175

[phy214720-bib-0016] Grassi, D. , Desideri, G. , Croce, G. , Tiberti, S. , Aggio, A. , & Ferri, C. (2009). Flavonoids, vascular function and cardiovascular protection. Current Pharmaceutical Design, 15, 1072–1084. 19355949 10.2174/138161209787846982

[phy214720-bib-0017] Grassi, D. , Desideri, G. , Di Giosia, P. , De Feo, M. , Fellini, E. , Cheli, P. , Ferri, L. , & Ferri, C. (2013). Tea, flavonoids, and cardiovascular health: Endothelial protection. American Journal of Clinical Nutrition, 98, 1660s–1666s. 10.3945/ajcn.113.058313 24172308

[phy214720-bib-0018] Grassi, D. , Mulder, T. P. J. , Draijer, R. , Desideri, G. , Molhuizen, H. O. F. , & Ferri, C. (2009). Black tea consumption dose‐dependently improves flow‐mediated dilation in healthy males. Journal of Hypertension, 27, 774–781. 19516176 10.1097/HJH.0b013e328326066c

[phy214720-bib-0019] Guasch‐Ferré, M. , Merino, J. , Sun, Q. , Fitó, M. , & Salas‐Salvadó, J. (2017). Dietary polyphenols, mediterranean diet, prediabetes, and type 2 diabetes: A narrative review of the evidence. Oxidative Medicine and Cellular Longevity, 2017, 1–16. 10.1155/2017/6723931. PMC5572601 28883903

[phy214720-bib-0020] Hagobian, T. A. , & Braun, B. (2006). Interactions between energy surplus and short‐term exercise on glucose and insulin responses in healthy people with induced, mild insulin insensitivity. Metabolism, 55, 402–408. 16483886 10.1016/j.metabol.2005.09.017

[phy214720-bib-0021] Hamburg, N. M. , McMackin, C. J. , Huang, A. L. , Shenouda, S. M. , Widlansky, M. E. , Schulz, E. , Gokce, N. , Ruderman, N. B. , Keaney, J. F. , & Vita, J. A. (2007). Physical inactivity rapidly induces insulin resistance and microvascular dysfunction in healthy volunteers. Arteriosclerosis, Thrombosis, and Vascular Biology, 27, 2650–2656. 10.1161/ATVBAHA.107.153288 PMC2596308 17932315

[phy214720-bib-0022] Hanson, R. L. , Pratley, R. E. , Bogardus, C. , Narayan, K. M. , Roumain, J. M. , Imperatore, G. , Fagot‐Campagna, A. , Pettitt, D. J. , Bennett, P. H. , & Knowler, W. C. (2000). Evaluation of simple indices of insulin sensitivity and insulin secretion for use in epidemiologic studies. American Journal of Epidemiology, 151, 190–198. 10645822 10.1093/oxfordjournals.aje.a010187

[phy214720-bib-0023] Hodgson, J. M. , & Croft, K. D. (2010). Tea flavonoids and cardiovascular health. Molecular Aspects of Medicine, 31, 495–502. 20837049 10.1016/j.mam.2010.09.004

[phy214720-bib-0024] Jing, Y. , Han, G. , Hu, Y. , Bi, Y. , Li, L. , & Zhu, D. (2009). Tea consumption and risk of type 2 diabetes: A meta‐analysis of cohort studies. Journal of General Internal Medicine, 24, 557–562. 19308337 10.1007/s11606-009-0929-5 PMC2669862

[phy214720-bib-0025] Jochmann, N. , Lorenz, M. , Av, K. , Martus, P. , Böhm, V. , Baumann, G. , Stangl, K. , & Stangl, V. (2008). The efficacy of black tea in ameliorating endothelial function is equivalent to that of green tea. British Journal of Nutrition, 99, 863–868. 10.1017/S0007114507838992 17916273

[phy214720-bib-0026] Kim, K. , Vance, T. M. , & Chun, O. K. (2016). Greater flavonoid intake is associated with improved CVD risk factors in US adults. British Journal of Nutrition, 115, 1481–1488. 10.1017/S0007114516000519 26931451

[phy214720-bib-0027] Knudsen, S. H. , Hansen, L. S. , Pedersen, M. , Dejgaard, T. , Hansen, J. , Hall, G. V. , Thomsen, C. , Solomon, T. P. J. , Pedersen, B. K. , & Krogh‐Madsen, R. (2012). Changes in insulin sensitivity precede changes in body composition during 14 days of step reduction combined with overfeeding in healthy young men. Journal of Applied Physiology, 113, 7–15. 22556394 10.1152/japplphysiol.00189.2011

[phy214720-bib-0028] Kooijman, M. , Thijssen, D. H. , de Groot, P. C. , Bleeker, M. W. , van Kuppevelt, H. J. , Green, D. J. , Rongen, G. A. , Smits, P. , & Hopman, M. T. (2008). Flow‐mediated dilatation in the superficial femoral artery is nitric oxide mediated in humans. Journal of Physiology, 586, 1137–1145. 10.1113/jphysiol.2007.145722 PMC2375653 18096601

[phy214720-bib-0029] Liu, C. Y. , Huang, C. J. , Huang, L. H. , Chen, I. J. , Chiu, J. P. , & Hsu, C. H. (2014). Effects of green tea extract on insulin resistance and glucagon‐like peptide 1 in patients with type 2 diabetes and lipid abnormalities: a randomized, double‐blinded, and placebo‐controlled trial. PLoS One, 9, e91163. 24614112 10.1371/journal.pone.0091163 PMC3948786

[phy214720-bib-0030] Loke, W. M. , Proudfoot, J. M. , Hodgson, J. M. , McKinley, A. J. , Hime, N. , Magat, M. , Stocker, R. , & Croft, K. D. (2010). Specific dietary polyphenols attenuate atherosclerosis in apolipoprotein e–knockout mice by alleviating inflammation and endothelial dysfunction. Arteriosclerosis, Thrombosis, and Vascular Biology, 30, 749–757. 10.1161/ATVBAHA.109.199687 20093625

[phy214720-bib-0031] Lorenz, M. , Rauhut, F. , Hofer, C. , Gwosc, S. , Muller, E. , Praeger, D. , Zimmermann, B. F. , Wernecke, K. D. , Baumann, G. , Stangl, K. , & Stangl, V. (2017). Tea‐induced improvement of endothelial function in humans: No role for epigallocatechin gallate (EGCG). Scientific Reports, 7, 2279. 28536463 10.1038/s41598-017-02384-x PMC5442103

[phy214720-bib-0032] Mangels, D. R. , & Mohler, E. R. (2017). Catechins as potential mediators of cardiovascular health. Arteriosclerosis, Thrombosis, and Vascular Biology, 37, 757–763. 10.1161/ATVBAHA.117.309048 28336557

[phy214720-bib-0033] Marena, S. , Montegrosso, G. , De Michieli, F. , Pisu, E. , & Pagano, G. (1992). Comparison of the metabolic effects of mixed meal and standard oral glucose tolerance test on glucose, insulin and C‐peptide response in healthy, impaired glucose tolerance, mild and severe non‐insulin‐dependent diabetic subjects. Acta Diabetologica, 29, 29–33. 1520903 10.1007/BF00572826

[phy214720-bib-0034] Matsuda, M. , & DeFronzo, R. A. (1999). Insulin sensitivity indices obtained from oral glucose tolerance testing: comparison with the euglycemic insulin clamp. Diabetes Care, 22, 1462–1470. 10480510 10.2337/diacare.22.9.1462

[phy214720-bib-0035] Michas, G. , Micha, R. , & Zampelas, A. (2014). Dietary fats and cardiovascular disease: Putting together the pieces of a complicated puzzle. Atherosclerosis, 234, 320–328. 24727233 10.1016/j.atherosclerosis.2014.03.013

[phy214720-bib-0036] Nagao, T. , Meguro, S. , Hase, T. , Otsuka, K. , Komikado, M. , Tokimitsu, I. , Yamamoto, T. , & Yamamoto, K. (2009). A catechin‐rich beverage improves obesity and blood glucose control in patients with type 2 diabetes. Obesity, 17, 310–317. 19008868 10.1038/oby.2008.505

[phy214720-bib-0037] Nosova, E. V. , Yen, P. , Chong, K. C. , Alley, H. F. , Stock, E. O. , Quinn, A. , Hellmann, J. , Conte, M. S. , Owens, C. D. , Spite, M. , & Grenon, S. M. (2014). Short‐term physical inactivity impairs vascular function. Journal of Surgical Research, 190, 672–682. 10.1016/j.jss.2014.02.001 PMC4096607 24630521

[phy214720-bib-0038] Park, C. S. , Kim, W. , Woo, J. S. , Ha, S. J. , Kang, W. Y. , Hwang, S. H. , Park, Y. W. , Kim, Y. S. , Ahn, Y. K. , Jeong, M. H. , & Kim, W. (2010). Green tea consumption improves endothelial function but not circulating endothelial progenitor cells in patients with chronic renal failure. International Journal of Cardiology, 145, 261–262. 19962201 10.1016/j.ijcard.2009.09.471

[phy214720-bib-0039] Park, J.‐H. , Bae, J.‐H. , Im, S.‐S. , & Song, D.‐K. (2014). Green tea and type 2 diabetes. Integrative Medicine Research, 3, 4–10. 28664072 10.1016/j.imr.2013.12.002 PMC5481694

[phy214720-bib-0040] Parry, S. A. , Smith, J. R. , Corbett, T. R. B. , Woods, R. M. , & Hulston, C. J. (2017). Short‐term, high‐fat overfeeding impairs glycaemic control but does not alter gut hormone responses to a mixed meal tolerance test in healthy, normal‐weight individuals. British Journal of Nutrition, 117, 48–55. 10.1017/S0007114516004475 28115026

[phy214720-bib-0041] Peace, A. , Van Mil, A. , Jones, H. , & Thijssen, D. H. J. (2018). Similarities and differences between carotid artery and coronary artery function. Current Cardiology Reviews, 14, 254–263. 30198437 10.2174/1573403X14666180910125638 PMC6300794

[phy214720-bib-0042] Peng, X. , Zhou, R. , Wang, B. , Yu, X. , Yang, X. , Liu, K. , & Mi, M. (2014). Effect of green tea consumption on blood pressure: a meta‐analysis of 13 randomized controlled trials. Scientific Reports, 4, 6251. 25176280 10.1038/srep06251 PMC4150247

[phy214720-bib-0043] Perez‐Jimenez, J. , Neveu, V. , Vos, F. , & Scalbert, A. (2010). Identification of the 100 richest dietary sources of polyphenols: An application of the Phenol‐Explorer database. European Journal of Clinical Nutrition, 64, S112–S120. 21045839 10.1038/ejcn.2010.221

[phy214720-bib-0044] Perneger, T. V. (1998). What's wrong with Bonferroni adjustments? British Medical Journal, 316, 1236. 9553006 10.1136/bmj.316.7139.1236 PMC1112991

[phy214720-bib-0045] Ponzo, V. , Goitre, I. , Fadda, M. , Gambino, R. , De Francesco, A. , Soldati, L. , Gentile, L. , Magistroni, P. , Cassader, M. , & Bo, S. (2015). Dietary flavonoid intake and cardiovascular risk: A population‐based cohort study. Journal of Translational Medicine, 13, 218. 26152229 10.1186/s12967-015-0573-2 PMC4494724

[phy214720-bib-0046] Ras, R. T. , Zock, P. L. , & Draijer, R. (2011). Tea consumption enhances endothelial‐dependent vasodilation; a meta‐analysis. PLoS One, 6, e16974. 21394199 10.1371/journal.pone.0016974 PMC3048861

[phy214720-bib-0047] Restaino, R. M. , Holwerda, S. W. , Credeur, D. P. , Fadel, P. J. , & Padilla, J. (2015). Impact of prolonged sitting on lower and upper limb micro‐ and macrovascular dilator function. Experimental Physiology, 100, 829–838. 25929229 10.1113/EP085238 PMC4956484

[phy214720-bib-0048] Rubenfire, M. , Rajagopalan, S. , & Mosca, L. (2000). Carotid artery vasoreactivity in response to sympathetic stress correlates with coronary disease risk and is independent of wall thickness. Journal of the American College of Cardiology, 36, 2192–2197. 11127460 10.1016/s0735-1097(00)01021-4

[phy214720-bib-0049] Schroeter, H. , Heiss, C. , Balzer, J. , Kleinbongard, P. , Keen, C. L. , Hollenberg, N. K. , Sies, H. , Kwik‐Uribe, C. , Schmitz, H. H. , & Kelm, M. (2006). (‐)‐Epicatechin mediates beneficial effects of flavanol‐rich cocoa on vascular function in humans. Proc Natl Acad Sci U S A, 103, 1024–1029. 16418281 10.1073/pnas.0510168103 PMC1327732

[phy214720-bib-0050] Skeaff, C. M. , & Miller, J. (2009). Dietary fat and coronary heart disease: Summary of evidence from prospective cohort and randomised controlled trials. Annals of Nutrition and Metabolism, 55, 173–201. 19752542 10.1159/000229002

[phy214720-bib-0051] Suliburska, J. , Bogdanski, P. , Szulinska, M. , Stepien, M. , Pupek‐Musialik, D. , & Jablecka, A. (2012). Effects of green tea supplementation on elements, total antioxidants, lipids, and glucose values in the serum of obese patients. Biological Trace Element Research, 149, 315–322. 22581111 10.1007/s12011-012-9448-z PMC3501173

[phy214720-bib-0052] Thijssen, D. H. J. , Black, M. A. , Pyke, K. E. , Padilla, J. , Atkinson, G. , Harris, R. A. , Parker, B. , Widlansky, M. E. , Tschakovsky, M. E. , & Green, D. J. (2011). Assessment of flow‐mediated dilation in humans: a methodological and physiological guideline. American Journal of Physiology ‐ Heart and Circulatory Physiology, 300, H2–H12. 20952670 10.1152/ajpheart.00471.2010 PMC3023245

[phy214720-bib-0053] Thijssen, D. H. J. , Bruno, R. M. , van Mil, A. , Holder, S. M. , Faita, F. , Greyling, A. , Zock, P. L. , Taddei, S. , Deanfield, J. E. , Luscher, T. , Green, D. J. , & Ghiadoni, L. (2019). Expert consensus and evidence‐based recommendations for the assessment of flow‐mediated dilation in humans. European Heart Journal, 40, 2534–2547. 31211361 10.1093/eurheartj/ehz350

[phy214720-bib-0054] Tinahones, F. J. , Rubio, M. A. , Garrido‐Sanchez, L. , Ruiz, C. , Gordillo, E. , Cabrerizo, L. , & Cardona, F. (2008). Green tea reduces LDL oxidability and improves vascular function. Journal of the American College of Nutrition, 27, 209–213. 18689551 10.1080/07315724.2008.10719692

[phy214720-bib-0055] Tudor‐Locke, C. , Craig, C. L. , Brown, W. J. , Clemes, S. A. , De Cocker, K. , Giles‐Corti, B. , Hatano, Y. , Inoue, S. , Matsudo, S. M. , Mutrie, N. , Oppert, J. M. , Rowe, D. A. , Schmidt, M. D. , Schofield, G. M. , Spence, J. C. , Teixeira, P. J. , Tully, M. A. , & Blair, S. N. (2011). How many steps/day are enough? For adults. International Journal of Behavioral Nutrition and Physical Activity, 8, 79. 10.1186/1479-5868-8-79 PMC3197470 21798015

[phy214720-bib-0056] Utzschneider, K. M. , Prigeon, R. L. , Faulenbach, M. V. , Tong, J. , Carr, D. B. , Boyko, E. J. , Leonetti, D. L. , McNeely, M. J. , Fujimoto, W. Y. , & Kahn, S. E. (2009). Oral disposition index predicts the development of future diabetes above and beyond fasting and 2‐h glucose levels. Diabetes Care, 32, 335–341. 18957530 10.2337/dc08-1478 PMC2628704

[phy214720-bib-0057] van Mil, A. C. , Hartman, Y. , van Oorschot, F. , Heemels, A. , Bax, N. , Dawson, E. A. , Hopkins, N. , Hopman, M. T. , Green, D. J. , Oxborough, D. L. , & Thijssen, D. H. (2017). Correlation of carotid artery reactivity with cardiovascular risk factors and coronary artery vasodilator responses in asymptomatic, healthy volunteers. Journal of Hypertension, 35, 1026–1034. 28129249 10.1097/HJH.0000000000001274

[phy214720-bib-0058] Vita, J. A. (2005). Polyphenols and cardiovascular disease: Effects on endothelial and platelet function. The American Journal of Clinical Nutrition, 81, 292S–297S. 15640493 10.1093/ajcn/81.1.292S

[phy214720-bib-0059] Walhin, J.‐P. , Richardson, J. D. , Betts, J. A. , & Thompson, D. (2013). Exercise counteracts the effects of short‐term overfeeding and reduced physical activity independent of energy imbalance in healthy young men. The Journal of Physiology, 591, 6231–6243. 24167223 10.1113/jphysiol.2013.262709 PMC3892474

[phy214720-bib-0060] WHO . (2017). Cardiovascular Diseases ‐ Key Facts. https://www.WHO.int/en/news‐room/fact‐sheets/detail/cardiovascular‐diseases‐(cvds). [Accessed 27th May 2019].

[phy214720-bib-0061] Widlansky, M. E. , Hamburg, N. M. , Anter, E. , Holbrook, M. , Kahn, D. F. , Elliott, J. G. , Keaney, J. F. Jr , & Vita, J. A. (2007). Acute EGCG supplementation reverses endothelial dysfunction in patients with coronary artery disease. Journal of the American College of Nutrition, 26, 95–102. 17536120 10.1080/07315724.2007.10719590 PMC3773609

[phy214720-bib-0062] Wilmot, E. G. , Edwardson, C. L. , Achana, F. A. , Davies, M. J. , Gorely, T. , Gray, L. J. , Khunti, K. , Yates, T. , & Biddle, S. J. H. (2012). Sedentary time in adults and the association with diabetes, cardiovascular disease and death: systematic review and meta‐analysis. Diabetologia, 55, 2895–2905. 22890825 10.1007/s00125-012-2677-z

[phy214720-bib-0063] Wu, A. H. , Spicer, D. , Stanczyk, F. Z. , Tseng, C. C. , Yang, C. S. , & Pike, M. C. (2012). Effect of 2‐month controlled green tea intervention on lipoprotein cholesterol, glucose, and hormone levels in healthy postmenopausal women. Cancer Prevention Research, 5, 393–402. 22246619 10.1158/1940-6207.CAPR-11-0407 PMC3777853

[phy214720-bib-0064] Yahya, H. M. , Day, A. , Lawton, C. , Myrissa, K. , Croden, F. , Dye, L. , & Williamson, G. (2016). Dietary intake of 20 polyphenol subclasses in a cohort of UK women. European Journal of Nutrition, 55, 1839–1847. 26210882 10.1007/s00394-015-1001-3

